# Perioperative anti-vascular endothelial growth factor agents treatment in patients undergoing vitrectomy for complicated proliferative diabetic retinopathy: a network meta-analysis

**DOI:** 10.1038/s41598-020-75896-8

**Published:** 2020-11-03

**Authors:** Dong-yue Wang, Xin-yu Zhao, Wen-fei Zhang, Li-hui Meng, You-xin Chen

**Affiliations:** 1 Department of Ophthalmology, Peking Union Medical College Hospital, Chinese Academy of Medical Sciences, Beijing, 100730 China; 2 Peking Union Medical College (PUMC), PUMC & Chinese Academy of Medical Sciences, Beijing, 100730 China; 3 Key Lab of Ocular Fundus Diseases, Chinese Academy of Medical Sciences, Beijing, 100730 China

**Keywords:** Eye diseases, Medical research

## Abstract

Currently, controversies regarding the optimal time-point of anti-vascular endothelial growth factor (VEGF) pretreatment before pars plana vitrectomy (PPV) for proliferative diabetic retinopathy (PDR) still exist. To clarify this, we conducted a network meta-analysis, 26 randomized controlled trials including 1806 PDR patients were included. Compared with the sham group, performing anti-VEGF injection at preoperative (Pre-Op) 6 to 14 days could significantly improve post-operative best-corrected visual acuity (BCVA) and decrease the incidence of recurrent vitreous hemorrhage (VH). Meanwhile, it could significantly reduce the duration of surgery. Performing anti-VEGF injection at Pre-Op more than 14 days, 6 to 14 days or 1 to 5 days could significantly reduce the incidence of intra-operative bleeding, while no significant benefit existed at the end of PPV (*P* > 0.05). No significant difference existed between all those strategies and sham group in reducing the rate of silicone oil tamponade. Based on currently available evidence, performing the anti-VEGF pretreatment at pre-operative 6 to 14 days showed best efficacy in improving post-operative BCVA, reducing the duration of surgery and incidence of recurrent VH, it also achieves satisfactory effect in reducing the incidence of intra-operative bleeding.

## Introduction

Despite the understanding and management of diabetes had evolved tremendously over the last decades, diabetic retinopathy (DR) is still one of the leading causes of legally blind and responsible for up to 4.8% of blindness globally^1^. Proliferative diabetic retinopathy (PDR) is the worst stage of DR and always complicated with vitreous hemorrhage (VH) and even tractional retinal detachment (TRD)^2^. These complications are major causes of severe visual damage in PDR patients and need timely surgical interventions^3,4^.

Pars plana vitrectomy (PPV) combined with anti-vascular endothelial growth factor (VEGF) agents injections had been widely accepted to be the standard management for PDR patients complicated with VH or TRD^5,6^. Our previous meta-analysis^2^ had confirmed the pretreatment of anti-VEGF agents before vitrectomy for patients with complicated PDR might achieve much smoother surgery and better visual rehabilitation, reduce the rate of early recurrent VH and accelerate its absorption.

However, numerous controversies still exist and could not be solved by traditional randomized controlled trials (RCT) or meta-analysis. Firstly, the optimum time-point for the injection of anti-VEGF agents remains controversial. Current RCT or traditional meta-analysis could only conclude a pairwise comparison among these strategies. For instance, several RCTs reported that pre-operative anti-VEGF injection 5 to 10 days before PPV was clinically superior to 1 to 3 days^7^, while no RCTs compared these time-points with anti-VEGF injection at the end of PPV or other time-points; Secondly, there are too many strategies for this anti-VEGF treatment reported by current studies, regarding different anti-VEGF agents, dosages and time-points^7,8^.

The network meta-analysis is a new form of data synthesis, which could combine both the direct and indirect evidence of current RCTs using statistical techniques, yielding an estimate of comparative efficacy^9,10^. Therefore, our network meta-analysis is performed to compare the efficacy of different perioperative time-points of anti-VEGF administration in patients undergoing PPV for complicated PDR, primarily looking at visual outcomes and recurrence of VH.

## Method

This study was performed in accordance with the guidelines given by the ‘Preferred Reporting Items for Systematic Reviews and Meta-Analysis (the ‘PRISMA’ statement)’^11^.

### Search strategy

The PubMed, Embase and the Cochrane Central Register of Controlled Trials were searched up to March 2020 to identify relevant RCTs. The following keywords or corresponding Medical Subject Headings (Mesh) were used: ‘Diabetic Retinopathy’, ‘anti-VEGF’, ‘bevacizumab’, ranibizumab’, ‘Randomized Controlled Trial ‘and ‘Vitrectomy’. The detailed electronic search strategy of PubMed was ((((((((((((((((((((((“Vascular Endothelial Growth Factors”[Mesh]) OR “KH902 fusion protein” [Supplementary Concept]) OR “aflibercept” [Supplementary Concept]) OR “Ranibizumab”[Mesh]) OR “Bevacizumab”[Mesh]) OR “pegaptanib” [Supplementary Concept]) OR Pegaptanib[Title/Abstract]) OR Bevacizumab[Title/Abstract]) OR Ranibizumab[Title/Abstract]) OR Aflibercept[Title/Abstract]) OR Conbercept[Title/Abstract]) OR Vascular Endothelial Growth Factors[Title/Abstract]) OR Macugen[Title/Abstract]) OR Avastin[Title/Abstract]) OR Lucentis[Title/Abstract]) OR Eylea[Title/Abstract]) OR anti-VEGF[Title/Abstract]))))) AND ((((“Vitrectomy”[Mesh]) OR Vitrectomy[Title/Abstract])))))))) AND ((((((((random*[Title/Abstract]) OR “Randomized Controlled Trial” [Publication Type])))))))) AND (((((((“Diabetic Retinopathy”[Mesh]) OR ((proliferative diabetic retinopathy[Title/Abstract]) OR PDR[Title/Abstract])))). The reference lists of the relevant articles were also manually examined to further identify potentially related RCTs. Only human studies published in English were considered.

### Selection criteria

Inclusion criteria of our analysis were (1) participants: complicated PDR, defined as TRD or non-resolving VH requiring surgical intervention; (2) intervention: diabetic PPV; (3) comparison: different time-points or regimens of intravitreal injection of anti-VEGF agents; (4) outcomes: at least one of the followings: BCVA (log MAR scale); intraoperative parameters ( including duration of surgery, intra-operative bleeding and silicone oil tamponade); postoperative parameters like recurrent VH; (5) Methodological criterion: RCTs.

Exclusion criteria were (1) patients with other intraocular diseases that may affect the vitreoretinal surgery, such as uveitis, proliferative vitreoretinopathy, retinal vascular disorders, congenital vitreoretinopathies and traumatic retinal detachment; (2) Other differences between case group and control group beside the application of anti-VEGF agents; (3) Insufficient data to estimate odds ratio (OR) or standardized mean difference (SMD); (4) animal studies or cadaver subjects; and (5) redundant publications.

### Data extraction and quality assessment

After consecutive procedures of screening titles and abstracts, obtaining the full text of each article and reviewing them, articles that met the eligibility criteria and fail the exclusion criteria were included. Two authors (X-yZ and D-yW) independently extracted and collated data using a standardized data collection protocol. The extracted data included study characteristics (including first author, publication year, study duration and treatment allocation), patient characteristics (mean age, gender ratio, mean baseline BCVA), interventions (anti-VEGF groups, intervention doses and usage), details of the surgical procedure, outcomes (change in BCVA, and postoperative evaluating parameters) and follow-up period. For updated publications with the same cohort of patients of the previous study, the data was extracted only once. The corresponding authors of the included articles would be contacted if the essential data were unavailable. Discrepancies were evaluated by kappa text and agreement was achieved by consensus. The Cochrane risk of bias assessment tool was used to assess the methodological quality and risk of bias^12^.

### Outcomes

The primary outcomes of interest were the post-operative best-corrected visual acuity (BCVA) at the final follow-up and the incidence of recurrent vitreous hemorrhage (VH). Secondary outcomes were the duration of surgery, the incidence of silicone oil tamponade and intra-operative bleeding.

### Data synthesis and statistical analysis

We separately used SMD for continuous outcomes and OR for dichotomous outcomes. The network meta-analysis was conducted with indirect and mixed comparisons in Stata version 14.0 (StataCorp. 2015. Stata Statistical Software: Release 14. College Station, TX: StataCorp LP.) through the mvmeta command, network command and self-programmed Stata routines. Cochran Q test and the *I*^2^ statistic were applied to assess the heterogeneity^13^. We use global inconsistency test by fitting design-by-treatment in the inconsistency model to evaluate the level of heterogeneity between direct and indirect estimates^14,15^. The local inconsistency was assessed using node-splitting method^15^. The loop-specific approach which assesses the difference between direct and indirect estimates for a specific comparison in the loop was also applied to check the inconsistency^16^. If the results of these inconsistency tests were acceptable (*P* > 0.05), the consistency model would be selected to compare all the regimens using direct and indirect data^17,18^. The rankograms, surface under the cumulative ranking (SUCRA) curves and the mean ranks were estimated to rank the intervention hierarchy of competing regimens in the network meta-analysis^19^. The higher SUCRA potentially represents superior efficacy. The publication bias of each outcome was clarified by the comparison-adjusted funnel plot. When heterogeneity or inconsistency was found substantial in any outcome (*P* < 0.05), both sensitivity analysis and subgroup analyses (publication year, sample size, etc.) would be conducted to identify the source of the heterogeneity. If the heterogeneity or inconsistency could not be eliminated, the pooling result of this specific outcome would be regarded as invalid.

## Results

### Study characteristics

**Figure 1:**
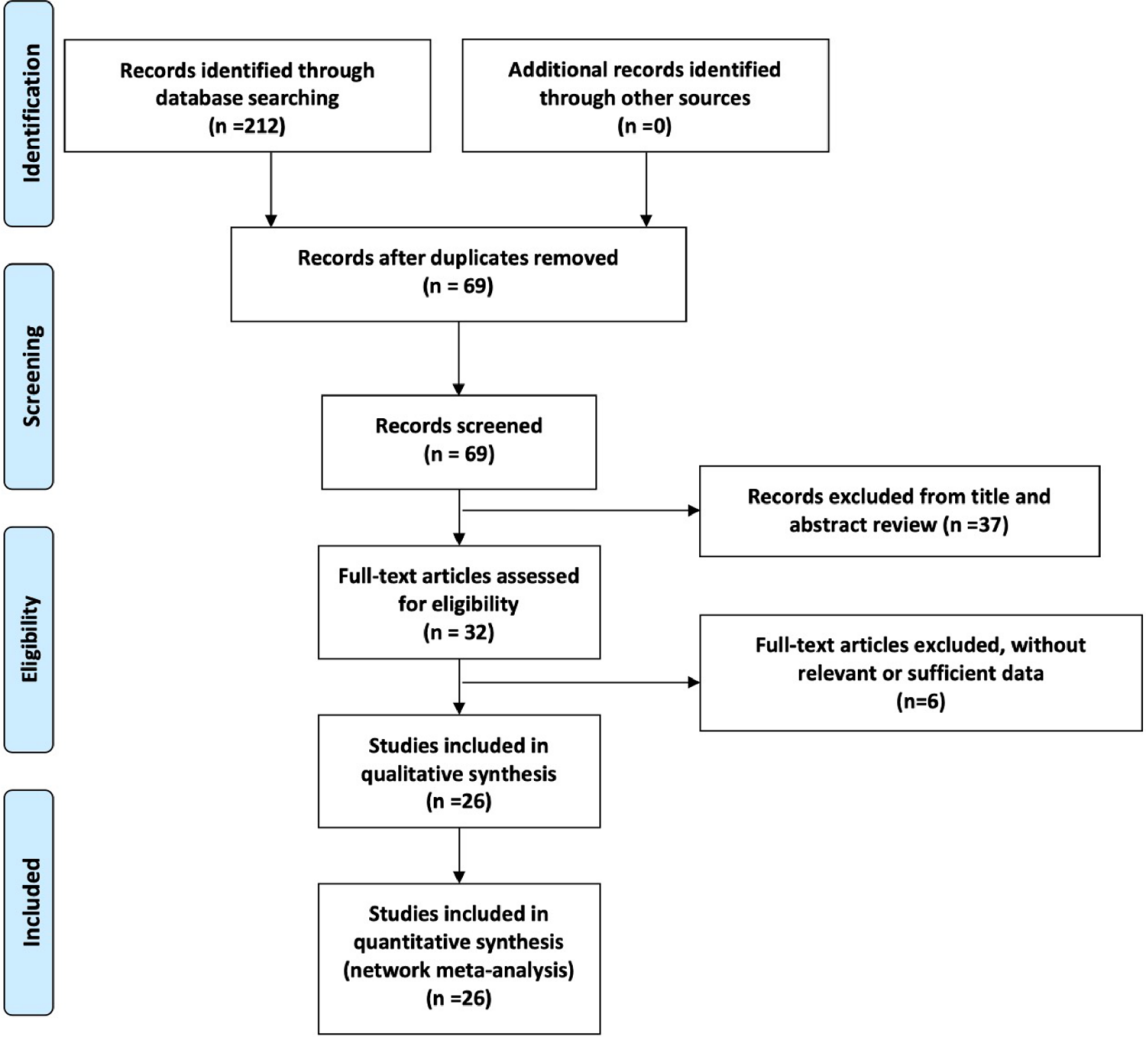
Flow chart describing the selecting process of included studies.

**Figure 2:**
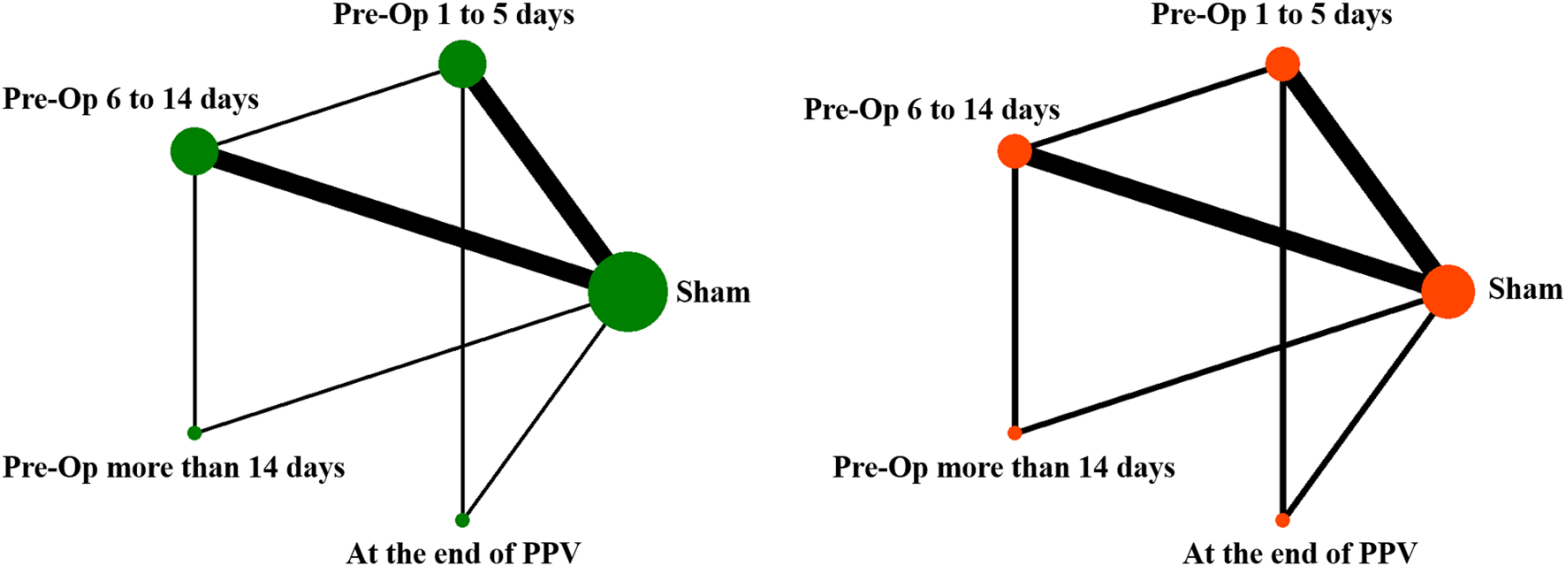
The network diagrams of all eligible comparisons for the primary outcomes of efficacy: (**A**) Post-operative best-corrected visual acuity; (**B**) Incidence of recurrent VH. *Pre-Op* pre-operative, *PPV* pars plana vitrectomy; *VH* vitreous hemorrhage. This figure was made by Xinyu Zhao and had got his permission to be published in this article.

We identified 212 citations by the initial search, then 32 potentially eligible articles were retrieved in full text after reviewing the titles and abstracts. Of these studies, 6 reports were excluded for irrelevant or insufficient data. Finally, 26 studies including 1802 PDR patients were included in our study, the detailed literature-exclusion procedures were described in Fig. 1. The inter-rater agreement was excellent between the investigators regarding eligibility (κ = 0.79). The main characteristics of these included studies were presented in Table 1. Five nodes regarding the timing of the anti-VEGF injection were included in our network meta-analysis, including pre-operative (Pre-Op) more than 14 days, 6 to 14 days, 1 to 5 days, at the end of PPV and sham injection (Fig. 2).

**Table 1:** Main characteristics of the included studies.

First author	Year	Study location	Design	Participants	Intervention	Group size	Patients (eyes)	Average age	Sex (M/F)	Outcomes	Follow-up
						Case/control		Case/control			
Rizzo et al.	2007	US	RCT	PDR	IVB, 1.25 mg, pre-Op 5–7 days versus Sham	11/11	22	52	NA	Complexity score, main-feasibility of surgery, BCVA	6 months
Pakzad-Vaezi et al.	2014	UK	RCT	PDR	IVB, 1.25 mg/0.05 mL versus IVR, 0.50 mg/0.05 mL	15/14	29	52.5	12/17	Total surgical time, TRD, Intraoperative bleeding, iatrogenic retinal breaks, use of endolaser and endodiathermy, silicone oil	In operation
Castillo et al.	2017	Mex	RCT	PDR	IVB, 2.5 mg/0.1 mL, pre-Op 1–3 days versus IVB, 2.5 mg/0.1 mL, pre-Op 5–10 days	73/65	126	54.9/57.4	70/68	BCVA, intraoperative surgery time, intraoperative complications, postoperative complications,	6 months
Arevalo et al.	2019	9 countries	RCT	PDR	IVB, 1.25 mg/0.1 mL, pre-Op 3–5 days versus Sham	102/112	224	59.5 ± 11.0/61.3 ± 10	116/68	Intraoperative bleeding, total surgical time, early postoperative VH, BCVA, endodiathermy applications, intraoperative retinal breaks, change in central macular thickness	12 months
Ahmadieh et al.	2009	Iran	RCT	PDR	IVB, 1.25 mg/0.05 mL, pre-Op 7 days versus sham	35/33	68	55.2 ± 11.1	34/34	Incidence of early post-vitrectomy hemorrhage, BCVA, IVB-related adverse events	1 months
Yang et al.	2015	China	RCT	PDR	IVC, 0.5 mg/0.05 mL, pre-Op 3 days versus sham	54/53	107	48.63/49.64	51/56	intraoperative bleeding, VH, BCVA, TRD, IOP, Endophthalmitis, Rubeosis, adverse events	3 months
Ahn et al.	2011	Korea	RCT	PDR	IVB, 1.25 mg/0.05 mL, pre-Op 1 to 14 days before PPV versus IVB, 1.25 mg/0.05 mL at the end of PPV versus sham	36/37/34	107	NA	NA	VH, time of vitreous clearing, BCVA	6 months
Lauro et al.	2009	Italy	RCT	PDR	IVB, 1.25 mg/0.1 mL, pre-Op 7 days versus IVB, 1.25 mg/0.1 mL, pre-Op 20 days versus Sham	24/24/24	72	NA	NA	Vitreous hemorrhage, configuration of retinal detachment, complexity surgery score, intraoperative bleeding, endodiathermy, iatrogenic break, relaxing retinotomy, silicone-oil tamponade, Surgical mean time	6 months
Modarres et al.	2009	Iran	RCT	PDR	IVB,2.5 mg/0.1 mL, pre-Op 3–5 days versus sham	22/18	40	5.8 ± 11.3/53.2 ± 11.7	NA	CS, BCVA, Number of endodiathermy applications, Backflush needle applications, Duration of surgery, VH	7 ± 3.6 months
Hernández-Da Mota et al.	2010	Mex	RCT	PDR	IVB, 1.25 mg/0.1 mL, pre-Op 2 days versus sham	20/20	40	55.7 ± 7.4/ 55.7 ± 9.9	24/16	BCVA, Intraoperative bleeding number of endodiathermy applications	6 months
Han et al.	2012	China	RCT	PDR	IVB, 1.25 mg, pre-Op 2 days versus sham	12/12	24	50.3/53.25	12/12	Number of vascular endothelial cells in NVMs, VEGF, HIF-1α	NA
Farahvash et al.	2011	Iran	RCT?	PDR	IVB, 1.25 mg/0.05 mL, pre-Op 7 days versus sham	18/17	35	58.5	18/17	Intraoperative complexity score, intraoperative bleeding, break formation, endodiathermy, CS, BCVA	7 months
Aleman et al.	2019	US	RCT	PDR	IVZ, 1.25 mg/0.05 mL, pre-Op 1–10 days versus IVB 1.25 mg/0.5 mL, pre-Op 1–10 days	82/91	206	58/55.8	91/82	BCVA, TRD, surgical time, Intraoperative and postoperative complications	6 months
Velazquez et al.	2018	Mex	RCT	PDR	A: IVB, 0.625 mg/0.025 mL, pre-Op 1–10 days; B: IVB 1.25 mg/0.05 mL, pre-Op 1–10 days; C: IVB 2.5 mg/0.1 mL, pre-Op 1–10 days	75/59/72	206	57.3/55.6/56.3	74/93	BCVA, TRD, intraoperative and postoperative complications	6 months
Comyn et al.	2017	UK	RCT	PDR	IVR, 0.5 mg/0.05 mL, pre-Op 7 days versus Sham	15/15	30	48.7/57.1	18/12	ETDRS BCVA, extend of TRD and Macular perfusion, surgery Time, surgery instrument usage, intraoperative haemorrhage, postoperative vitreous cavity haemorrhage	3 months
Hattori et al.	2010	Japan	RCT	PDR	IVB, 0.53 ± 0.39 (0.16–1.25), pre-Op 3 days versus Sham	12/40	52	59.1 ± 9.4	NA	VEGF concentration, Numbers of intraoperative coagulation spots	NA
Manabe et al.	2015	Japan	RCT	PDR	IVB, 0.16 mg/0.05 mL, pre-Op 1 day versus sham	32/34	66	59.9 ± 11.8/ 59.2 ± 12.9	54/12	VH, numbers of intraoperative laser, endodiathermy, concentration of VEGF	1 months
Su et al.	2016	China	RCT	PDR	IVC, 0.5 mg/0.05 mL, pre-Op 7 days versus sham	18/18	36	NA	NA	BCVA, intraoperative bleeding, Endodiathermy, Iatrogenic break, Silicone oil, Surgical mean time	6 weeks
Zaman et al.	2013	Pakistan	RCT	PDR	IVB, 1.25 mg /0,05 mL, pre-Op 7 days versus sham	30/24	54	52.07 ± 5.54	32/22	BCVA, postoperative complication, VH	6 months
Jeon et al.	2012	Korea	RCT	PDR	IVB, 1.25 mg/0.05 mL, pre-Op 1-day versus IVB 1.25 mg/0.05 mL, pre-Op 7 days	15/15	30	58,71 ± 9.77/ 55.83 ± 10.67	19/11	VEGF, IL-6, IL-8, TGF-β2, IL-2, TNF-α	1 day
Li et al.	2015	China	RCT	PDR	IVB, 1.25 mg/0.05 mL, pre-Op 5 days versus IVB, 1.25 mg/0.05 mL , pre-Op > 14 days versus Sham	23/11/19	68	48.9 ± 11.2/53.9 ± 8.5	29/24	Vitreous VEGF, bFGF, fibrosis	NA
Lucena et al.	2009	USA	RCT	PDR	IVB 1.25 mg/0.05 mL, pre-Op 14 days versus sham	10/10	20	NA	10/10	Amount of intraocular haemorrhage,	NA
Zhou et al.	2018	China	RCT	PDR	IVC, 0.5 mg, pre-Op 7 days versus sham	9/9	16	51.69 ± 8.5	14/11	BCVA, VEGF, PIGF	3 months
Li et al.	2020	China	RCT	PDR	IVC, 0.5 mg/0.05 mL, pre-Op 7 days versus IVC, 0.5 mg/0.05 mL, pre-Op 14 days versus sham	20/20/20	60	50.6 ± 5.6	32/28	CS, intraoperative bleeding, VEGF concentrations, total surgical time	NA
Gao et al.	2020	China	RCT	PDR	IVC, 0.5 mg/0.05 mL, pre-Op 3–5 days versus IVC, 0.5 mg/0.05 mL, end of surgery	34/35	69	50.76 ± 13.47/53.97 ± 14.76	30/39	BCVA, IOP, intraoperative bleeding, surgery duration, postoperative follow-up	6 months
Cui et al.	2018	China	RCT	PDR	IVC, 0.5 mg/0.05 mL, pre-Op 3–7 days versus IVR, 0.5 mg/0.05 mL, pre-Op 3–7 days	20/19	39	60.74 ± 2.63/55.28 ± 5.16	24/15	BCVA, operation time, incidence of iatrogenic retinal breaks, endodiathermy rate, and silicone oil tamponade, vitreous clearing time, intraoperative and postoperative bleeding	6 months

*BCVA* best corrected visual acuity, *CS* complexity score, *IOP* intraocular pressure, *IVB* intravitreal injection of Bevacizumab, *IVC* intravitreal injection of conbercept, *IVR* intravitreal injection of ranibizumab, *NVM* neovascular membrane, *NA* not available, *PDR* photodynamic therapy, *RCT* randomized controlled trial, *TRD* tractional retinal detachment, *VEGF* vascular endothelial growth factor, *VH* vitreous hemorrhage.

In general, most of these studies (25 of 26) were judged to have an unclear risk of bias (Suppl. 1, 2), none of these studies had evidence of a definite high risk in any item.

### Primary outcomes

**Figure 3:**
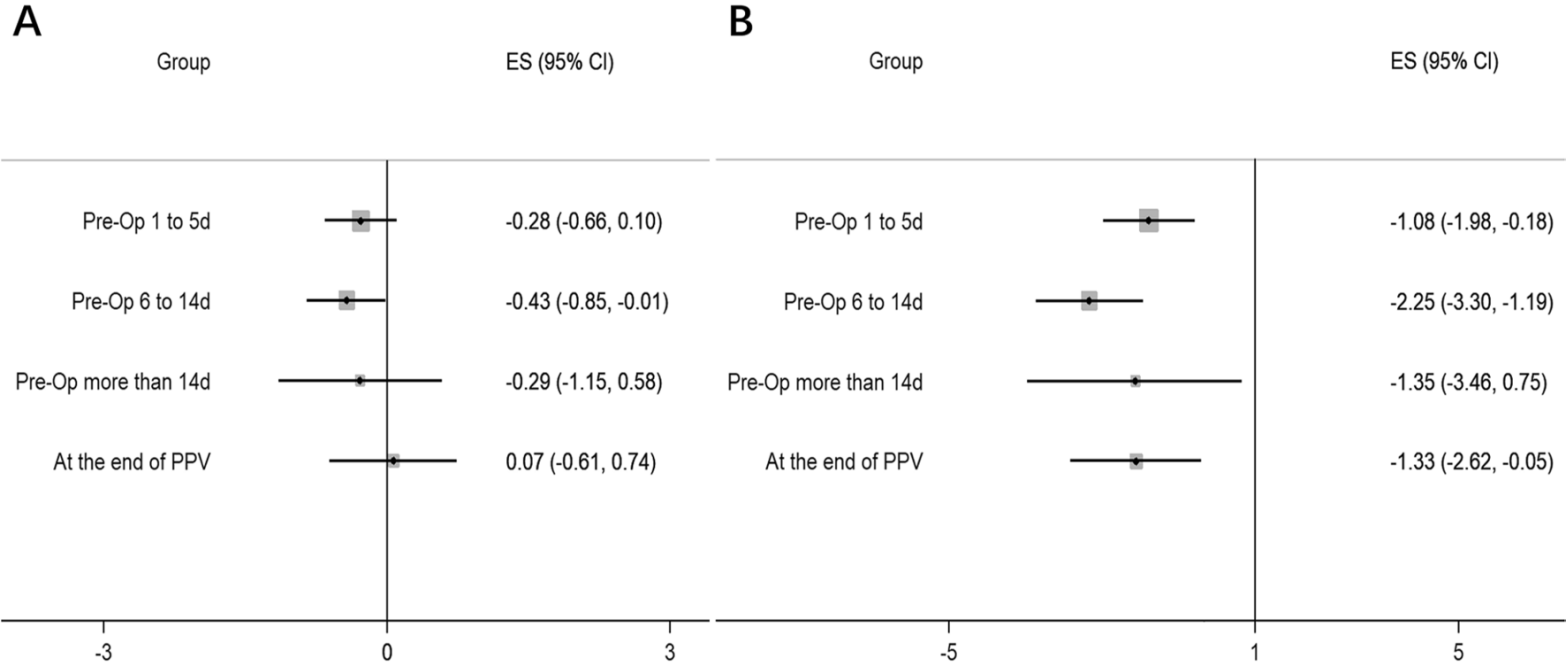
Network meta-analysis of different time-points of perioperative anti-VEGF treatment compared with sham treatment for the primary outcomes: (**A**) Post-operative BCVA; (**B**) Incidence of recurrent VH. *Pre-Op* pre-operative, *PPV* pars plana vitrectomy, *VH* vitreous hemorrhage, *BCVA* best-corrected visual acuity.

The network diagrams of all eligible comparisons for the primary outcomes are presented in Fig. 2 and the results of network meta-analysis were shown in Fig. 3. The mean ranking based on SUCRA curves of the primary outcomes were shown in Table 2, a higher SUCRA potentially means superior efficacy. The detailed results of head-to-head comparisons were provided in Table 3.

**Table 2:** The estimated mean ranking based on surface under the cumulative ranking (SUCRA) curves of all primary and secondary outcomes in the network meta-analysis.

Ranking	Post-Op BCVA	SUCRA	Duration of surgery	SUCRA	Recurrent VH	SUCRA	Silicone oil tamponade	SUCRA	Intra-Op bleeding	SUCRA
Best	Pre-Op 6 to 14 days	80.3	Pre-Op 6 to 14 days	83.7	Pre-Op 6 to 14 days	90.9	Pre-Op more than 14 days	82.9	Pre-Op more than 14 days	79.2
2nd	Pre-Op 1 to 5 days	64	Pre-Op more than 14 days	76.2	At the end of PPV	56.2	Pre-Op 6 to 14 days	60.5	Pre-Op 6 to 14 days	74.6
3rd	Pre-Op more than 14 days	59.1	Pre-Op 1 to 5 days	40.5	Pre-Op more than 14 days	55.4	Sham	38.3	Pre-Op 1 to 5 days	55.5
4th	At the end of PPV	23.4	Sham	39.7	Pre-Op 1 to 5 days	44.1	At the end of PPV	39	At the end of PPV	27.5
5th	Sham	23.2	At the end of PPV	10	Sham	3.4	Pre-Op 1 to 5 days	29.4	Sham	13.4

A higher SUCRA potentially means superior efficacy or safety.

*BCVA* best-corrected visual acuity, *Pre-Op* pre-operative, *PPV* pars plana vitrectomy, *SUCRA* surface under the cumulative ranking.

**Table 3:**
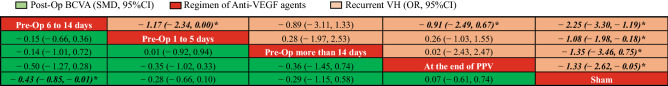
Head-to-head comparisons for primary outcomes of different time-points of perioperative anti-VEGF treatment.

The bold items mean *P* < 0.05.

*95% CI* 95% confidence interval, *BCVA* best-corrected visual acuity, *OR* odds risk, *Pre-Op* pre-operative, *PPV* pars plana vitrectomy, *SMD* standardized mean difference, *VEGF* vascular endothelial growth factor, *VH* vitreous hemorrhage.

Thirteen RCTs involving 889 patients provide adequate data for the primary outcome of post-operative BCVA and incidence of recurrent VH, the most effective time-point was estimated to be Pre-Op 6 to 14 days (Table 2). Compared with the sham group, performing anti-VEGF injection at Pre-Op 6 to 14 days could significantly improve post-operative BCVA (SMDs = − 0.43, 95% credible interval [CI]: − 0.85 to − 0.01, *P* < 0.05, Fig. 3) and decrease the incidence of recurrent VH (OR = − 2.25, 95% CI: − 3.3 to − 1.19, *P* < 0.05). Meanwhile, performing the anti-VEGF injection at the other three time-points could also significantly reduce the incidence of recurrent VH (*P* < 0.05), while no significant difference existed for post-operative BCVA when compared with the sham group (*P* > 0.05).

### Secondary outcomes

**Figure 4:**
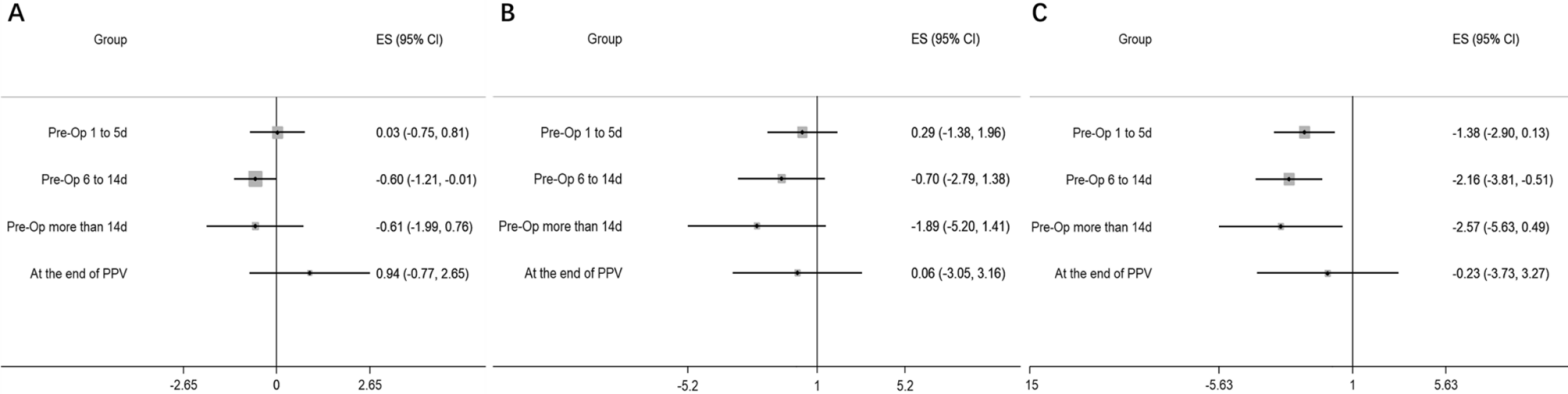
Network meta-analysis of different time-points of perioperative anti-VEGF treatment compared with sham treatment for the secondary outcomes: (**A**) Duration of surgery; (**B**) Rate of silicone oil tamponade; (**C**) Incidence of intra-operative bleeding. *Pre-Op* pre-operative, *PPV* pars plana vitrectomy.

The results of network meta-analysis were shown in Fig. 4. The corresponding mean ranking based on SUCRA curves was also listed in Table 2, a higher SUCRA potentially means superior efficacy. For all the secondary outcomes, detailed results of head-to-head comparisons were provided in Suppl. 3, 4, 5.

Eleven RCTs involving 762 patients reported the duration of surgery and Pre-Op 6 to 14 days was estimated to be the most effective strategy (Table 2). Compared with the sham group, Pre-Op 6 to 14 days could significantly reduce the duration of surgery (SMDs = − 0.60, 95% CI: − 1.21 to − 0.01, *P* < 0.05, Fig. 4), while no statistical difference existed between other time-points (*P* > 0.05).

Ten RCTs involving 715 patients describe the rate of silicone oil tamponade, Pre-Op more than 14 days was estimated to have the highest SUCRA ranking (Table 2), while there was no significant difference between all those strategies and sham group (*P* > 0.05, Fig. 4).

Eleven RCTs involving 791 patients evaluated the incidence of intra-operative bleeding. The network meta-analysis showed that Pre-Op more than 14 days achieved the highest SUCRA ranking (Table 2). Compared with the sham group, performing anti-VEGF injection at Pre-Op more than 14 days, 6 to 14 days or 1 to 5 days could significantly reduce the incidence of intra-operative bleeding (*P* < 0.05, Fig. 4), while conducting anti-VEGF injection at the end of PPV could not achieve any significant benefit (*P* > 0.05).

### Subgroup analysis

The subgroup SUCRA analysis was then conducted regarding the detailed interventions in each study, including different agents, dosages and time-points. The corresponding mean ranking based on SUCRA curves was listed in Table 4.

**Table 4:** The estimated mean ranking based on surface under the cumulative ranking (SUCRA) curves of the subgroup analysis.

Ranking	Post-Op BCVA		SUCRA	Duration of surgery	SUCRA	Recurrent VH	SUCRA	Silicone oil tamponade	SUCRA	Intra-Op bleeding	SUCRA
Best	IVB, 2.5 mg 6–14 days		91.9	IVC, 0.5 mg, 6–14 days	83.8	IVB, 2.5 mg, 6–14 days	88.1	IVB, 1.25 mg, > 14 days	75.4	IVB, 2.5 mg, 1–5 days	72.9
2nd	IVB, 2.5 mg, 1–5 d		81.2	IVB, 1.25 mg, 1–5 days	71.5	IVB, 2.5 mg, 1–5 days	73.5	IVB, 1.25 mg, 6–14 days	62.4	IVB, 1.25 mg, > 14 days	65.3
3rd	IVC, 0.5 mg, 6–14 d		57.6	IVB, 1.25 mg, 6–14 days	70.1	IVB, 1.25 mg, 6–14 days	62.1	IVB, 1.25 mg, 1–5 days	61.6	IVB, 1.25 mg, 6–14 days	62.8
4th	IVC, 0.5 mg, 1–5 days		54.5	IVB, 1.25 mg, > 14 days	68.3	IVC, 0.5 mg, end of surgery	59.7	IVB, 1.25 mg, at the end of PPV	50.5	IVC, 0.5 mg, 1–5 days	57.9
5th	IVB, 1.25 mg, 6–14 days		51.5	IVB, 2.5 mg, 6–14 days	53.2	IVC, 0.5 mg, 1–5 days	46.1	IVR, 0.5 mg, 7–14 days	50.4	IVR, 0.5 mg, 7–14 days	57.2
6th	IVC, 0.5 mg, at end of PPV		48.7	IVR, 0.5 mg, 7–14 days	49.8	IVB, 1.25 mg, > 14 days	43.6	Sham	49.1	IVC, 0.5 mg, 6–14 days	55.1
7th	IVB, 1.25 mg, > 14 days		45.6	IVB, 2.5 mg, 1–5 days	48.2	IVB, 1.25 mg, at the end of PPV	34.4	IVB, 2.5 mg, 1–5 days	45.5	IVR, 0.5 mg, 1–5 days	47.2
8th	IVB, 1.25 mg, 1–5 days		35.3	Sham	45.3	IVB, 1.25 mg, 1–5 days	29.5	IVC, 0.5 mg, at the end of PPV	37.8	IVC, 0.5 mg, at the end of PPV	37.4
9th	IVR, 0.5 mg, 1–5 days		34.7	IVR, 0.5 mg, 1–5 days	31.2	Sham	13	IVR, 0.5 mg, 1–5 days	34.9	IVB, 1.25 mg, 1–5 days	26.3
10th	Sham		29.9	IVC, 0.5 mg, 1–5 days	20.8	*IVR, 0.5 mg, 1–5 days*	NA	IVC, 0.5 mg, 1–5 days	32.2	Sham	17.8
11th	IVB, 1.25 mg, at the end of PPV		19	IVC, 0.5 mg, at the end of PPV	7.8	*IVC, 0.5 mg, 6–14 days*	NA	*IVB, 2.5 mg, 6–14 days*	NA	*IVB, 2.5 mg, 6–14 days*	NA
12th	*IVR, 0.5 mg, 7–14 days*		NA	*IVB, 1.25 mg, at the end of PPV*	NA	*IVR, 0.5 mg, 7–14 days*	NA	*IVC, 0.5 mg, 6–14 days*	NA	*IVB, 1.25 mg, at the end of PPV*	NA

Italic means the data was unobtainable or could not be included in the main closed loop; A higher SUCRA potentially means superior efficacy or safety.

*BCVA* best-corrected visual acuity, *IVB* intravitreal injection of Bevacizumab, *IVC* intravitreal injection of conbercept, *IVR* intravitreal injection of ranibizumab, *NA* not available, *Pre-Op* pre-operative, *Post-Op* post-operative, *PPV* pars plana vitrectomy, *SUCRA* surface under the cumulative ranking, *VH* vitreous hemorrhage.

### Inconsistency and heterogeneity

Global inconsistency, local inconsistency or heterogeneity were not significant between evidence derived from direct and indirect comparisons in both of the primary and secondary outcomes (*P* > 0.05). The corresponding comparison-adjusted funnel plots also showed no evidence of asymmetry (*P* > 0.05).

## Discussion

This analysis is a comprehensive network meta-analysis in evaluating the efficacy of different time-points of perioperative anti-VEGF injection for patients undergoing vitrectomy for complicated PDR. The results of our study indicated that anti-VEGF injection at pre-operative 6 to 14 days showed the best efficacy in improving post-operative BCVA, reducing the duration of surgery and incidence of recurrent VH, it also achieves satisfactory effect in reducing the incidence of intra-operative bleeding. Additionally, the general efficacy ranking of each detailed regimen was achieved for reference. More importantly, our study provides a solid reference for the current most concerned controversies mentioned in the introduction.

The purpose of perioperative anti-VEGF injection is to induce the regression of retinal neovascularization (RNV), decrease the intra-operative bleeding, and facilitate easier fibrovascular membrane dissection and smoother vitreoretinal surgery. Some authors suggested performing the injection with an interval of more than 14 days^20,21^, in order to make full use of anti-VEGF agents and induce the complete regression of RNV. While other expressed their concerns about the formation or aggravation of tractional retinal detachment (TRD) associated with progressive fibrosis of fibrovascular membrane following the pretreatment of anti-VEGF agents^22,23^, so they suggested performing the injection with a short interval like 1 to 3 days^6,24^. Russo et al.^25^ studied the incidence of tractional macular detachment following pre-vitrectomy anti-VEGF injection and showed that a longer period between the injection and the surgery increases the incidence of tractional macular detachment; in particular, when anti-VEGF injection was given within 6 days from PPV, tractional macular detachment happened in 2.7% of cases, when the injection was given more than 10 days before vitrectomy, rate of TMD increased to 56%.

For the postoperative BCVA, numerous factors might be associated with it, like the history of TRD, surgical trauma, recurrent VH, silicone oil tamponade, diabetic macular edema. Although it was “barely” significant (SMDs = − 0.43, 95% CI: − 0.85 to − 0.01), the pooling results of our study indicated that only performing anti-VEGF injection at Pre-Op 6 to 14 days could significantly improve post-operative BCVA compared with the sham group, which were also supported by the corresponding head-to-head comparisons^21^. So was the duration of surgery, only performing the injection at Pre-Op 6 to 14 days could significantly reduce the operative time compared with the sham group, which might mean easier and smoother surgery. While our study showed that these pretreatments could not significantly reduce the incidence of silicone oil tamponade, which is standard procedure for TRD and last resort for unstoppable intra-operative bleeding.

Routinely diabetic PPV without anti-VEGF pretreatment was always troublesome by intra-operative bleeding. Firstly, hemorrhages make it difficult to perform the delamination and segmentation of the fibrovascular tissue, they usually adhere tightly to retina surface, the removal of these tissues has high risk of iatrogenic retinal breaks^26^; Secondly, continued intra-operative bleeding may impede adequate endophotocoagulation as poor visualization, increasing the risk of rubeosis iridis and subsequently neovascular glaucoma after surgery; Additionally, difficult-to-control bleeding during surgery wastes plenty of time, which might cause other complications like corneal opacification and poor visualization of the surgical field^27^, all these may result in poor surgical outcome. Our studies showed that pretreatment of anti-VEGF could all significantly reduce the incidence of intra-operative bleeding, Pre-Op more than 14 days achieved the highest SUCRA ranking, while conducting anti-VEGF injection at the end of PPV could not achieve any beneficial effect. It is understandable as anti-VEGF agents need time to take effect, longer interval equals to better regression of NV and absorption of hemorrhages.

Recurrent VH after PPV for PDR is the major concern for both patients and surgeons, with a reported incidence up to 75%^28^. It might greatly jeopardize patient’s expectations, prevents clear fundus examination and further laser therapy. The source of early and late postoperative recurrent VH were different, early recurrent VH was associated with dissection of fibrovascular membranes, recurrent bleeding from initial bleeding site, surgically injured retinal tissue and increased vascular permeability^5,6^ while recurrent neovascularization was believed to be the crucial cause in late recurrent VH and RD^29^. The pooling results of our study indicated that performing the anti-VEGF injection at all the four time-points could achieve a significantly lower incidence of recurrent VH compared with the sham group, also leading by Pre-Op 6 to 14 days. However, anti-VEGF agents could only provide complete VEGF blockade for about 4 weeks and almost all the amount of anti-VEGF agents injected preoperatively would be removed during vitrectomy, panretinal photocoagulation should be done adequately during and post-surgery to prevent recurrent neovascularization and reduce the incidence of late recurrent VH and postoperative recurrent RD.

Our study still has several limitations. (1) Our findings are achieved through direct and indirect comparisons in a network meta-analysis. Although this method is widely accepted with better statistical precision^30^, it could not substitute results from large-scale RCTs; (2) Although we conducted the subgroup analysis regarding different agents, dosages and time-points, they included too many different regimens and it was difficult to achieve an universally applicable conclusion, the results might just give some hints like a higher dosage of anti-VEGF could achieve better outcomes than traditional dosage ; (3) Only RCTs published in English were considered.

## Conclusion

In summary, our study suggests that performing the anti-VEGF pretreatment at pre-operative 6 to 14 days showed the best efficacy in improving post-operative BCVA, reducing the duration of surgery and incidence of recurrent VH, it also achieves satisfactory effect in reducing the incidence of intra-operative bleeding.

## Supplementary information

Supplementary Information.

## Abbreviations

95% CI: 95% Confidence interval
BCVA: Best-corrected visual acuity
CS: Complexity score
DR: Diabetic retinopathy
ETDRS: Early treatment diabetic retinopathy study
NVM: Neovascular membrane
OR: Odds ratio
PPV: Pars Plana vitrectomy
PDR: Proliferative diabetic retinopathy
Pre-Op: Pre-operative
Post-Op: Post-operative
RCT: Randomized controlled trials
RD: Retinal detachment
RNV: Retinal neovascularization
Intra-Op: Intra-operative
SMD: Standardized mean difference
SUCRA: Surface under the cumulative ranking
TRD: Tractional retinal detachment
VEGF: Vascular endothelial growth factor
VA: Visual acuity
VH: Vitreous hemorrhage
