# Supplementary material for: Perioperative anti-vascular endothelial growth factor agents treatment in patients undergoing vitrectomy for complicated proliferative diabetic retinopathy: a network meta-analysis
Source: Sci Rep. 2020 Nov 3;10:18880. doi: 10.1038/s41598-020-75896-8 (PMC7641141; doi:10.1038/s41598-020-75896-8)
Supplement: Supplementary file 1 — Supplementary Information. [file 41598_2020_75896_MOESM1_ESM.docx]

**Title:** **Perioperative Anti-Vascular Endothelial Growth Factor Agents Treatment in Patients Undergoing Vitrectomy for Complicated Proliferative Diabetic Retinopathy：A Network Meta-Analysis**

**Authors:** Dong-yue Wang^1,2^, Xin-yu Zhao^1,3*^ M.D., Wen-fei Zhang^1,3^ M.D., Li-hui Meng^1,2^ M.D., You-xin Chen^1,3*^ M.D., Ph.D.

**Affiliations:**

1 Department of Ophthalmology, Peking Union Medical College Hospital, Chinese Academy of Medical Sciences, Beijing 100730, China

2 Peking Union Medical College (PUMC), PUMC & Chinese Academy of Medical Sciences, Beijing, 100730, China.

3 Key Lab of Ocular Fundus Diseases, Chinese Academy of Medical Sciences, Beijing 100730, China

***Co-corresponding Author:**

**Xin-yu Zhao** M.D
Department of Ophthalmology

Peking Union Medical College Hospital

Chinese Academy of Medical Sciences

Beijing 100730, China
Tel: +86-13121516170

E-mail: zhaoxinyu@pumch.cn

**You-xin Chen** M.D., Ph.D. Professor
Department of Ophthalmology

Peking Union Medical College Hospital

Chinese Academy of Medical Sciences

Beijing 100730, China
Tel: +86-13801025972

E-mail: ChenYX@pumch.cn

Supplement 1. Risk of bias graph for the included studies.


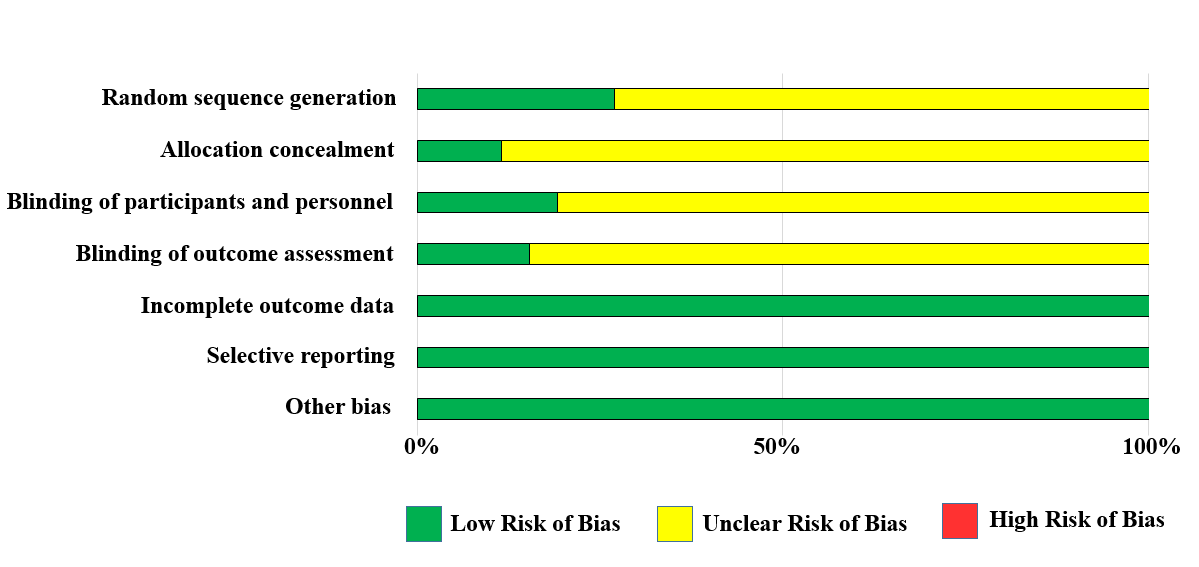


Supplement 2. Risk of bias summary graph for the included studies.

| **No.** | **Study** | **Random sequence generation** | **Allocation concealment** | **Blinding of participants and personnel** | **Blinding of outcome assessment** | **Incomplete outcome data** | **Selective reporting** | **Other bias** |
| --- | --- | --- | --- | --- | --- | --- | --- | --- |
| 1 | Rizzo et al. 2007 | Low risk | Unclear | Unclear | Unclear | Low risk | Low risk | Low risk |
| 2 | Pakzad-Vaezi et al. 2014 | Low risk | Low risk | Unclear | Unclear | Low risk | Low risk | Low risk |
| 3 | Castillo et al. 2017 | Unclear | Unclear | Unclear | Unclear | Low risk | Low risk | Low risk |
| 4 | Arevalo et al. 2019 | Low risk | Low risk | Low risk | Low risk | Low risk | Low risk | Low risk |
| 5 | Ahmadieh et al. 2009 | Low risk | Unclear | Low risk | Unclear | Low risk | Low risk | Low risk |
| 6 | Yang et al. 2015 | Unclear | Unclear | Unclear | Unclear | Low risk | Low risk | Low risk |
| 7 | Ahn et al. 2011 | Low risk | Unclear | Unclear | Unclear | Low risk | Low risk | Low risk |
| 8 | Lauro et al. 2009 | Unclear | Unclear | Unclear | Unclear | Low risk | Low risk | Low risk |
| 9 | Modarres et al. 2009 | Unclear | Unclear | Low risk | Unclear | Low risk | Low risk | Low risk |
| 10 | Hernández-Da Mota et al. 2010 | Unclear | Unclear | Unclear | Unclear | Low risk | Low risk | Low risk |
| 11 | Han et al. 2012 | Unclear | Unclear | Unclear | Unclear | Low risk | Low risk | Low risk |
| 12 | Farahvash et al. 2011 | Unclear | Unclear | Unclear | Unclear | Low risk | Low risk | Low risk |
| 13 | Aleman et al. 2019 | Unclear | Low risk | Unclear | Unclear | Low risk | Low risk | Low risk |
| 14 | Velazquez et al. 2018 | Low risk | Unclear | Low risk | Unclear | Low risk | Low risk | Low risk |
| 15 | Comyn et al. 2017 | Unclear | Unclear | Unclear | Unclear | Low risk | Low risk | Low risk |
| 16 | Hattori et al. 2010 | Unclear | Unclear | Unclear | Unclear | Low risk | Low risk | Low risk |
| 17 | Manabe et al. 2015 | Low risk | Unclear | Low risk | Unclear | Low risk | Low risk | Low risk |
| 18 | Su et al. 2016 | Unclear | Unclear | Unclear | Unclear | Low risk | Low risk | Low risk |
| 19 | Zaman et al. 2013 | Unclear | Unclear | Unclear | Unclear | Low risk | Low risk | Low risk |
| 20 | Jeon et al. 2012 | Unclear | Unclear | Unclear | Unclear | Low risk | Low risk | Low risk |
| 21 | Li et al. 2015 | Unclear | Unclear | Unclear | Unclear | Low risk | Low risk | Low risk |
| 22 | Lucena et al. 2009 | Unclear | Unclear | Unclear | Low risk | Low risk | Low risk | Low risk |
| 23 | Zhou et al. 2018 | Unclear | Unclear | Unclear | Unclear | Low risk | Low risk | Low risk |
| 24 | Li et al. 2020 | Unclear | Unclear | Unclear | Unclear | Low risk | Low risk | Low risk |
| 25 | Gao et al. 2020 | Unclear | Unclear | Unclear | Low risk | Low risk | Low risk | Low risk |
| 26 | Cui et al. 2018 | Unclear | Unclear | Unclear | Unclear | Low risk | Low risk | Low risk |

Supplement 3. The details of head-to-head comparisons of duration of surgery.

Regimen of anti-VEGF agents (SMD, 95%CI)

| **Pre-Op 6 to 14d** | -0.01 (-1.38,1.36) | 0.64 (-0.25,1.52) | ***0.60 (0.01,1.21)**** | 1.54 (-0.22,3.30) |
| --- | --- | --- | --- | --- |
| 0.01 (-1.36,1.38) | **Pre-Op more than 14d** | 0.65 (-0.90,2.19) | 0.61 (-0.76,1.99) | 1.55 (-0.62,3.72) |
| -0.64 (-1.52,0.25) | -0.65 (-2.19,0.90) | **Pre-Op 1 to 5d** | -0.03 (-0.81,0.75) | 0.91 (-0.61,2.43) |
| ***-0.60 (-1.21,-0.01)**** | -0.61 (-1.99,0.76) | 0.03 (-0.75,0.81) | **Sham** | 0.94 (-0.77,2.65) |
| -1.54 (-3.30,0.22) | -1.55 (-3.72,0.62) | -0.91 (-2.43,0.61) | -0.94 (-2.65,0.77) | **At the end of PPV** |

95% CI=95% Confidence Interval; Pre-Op=Pre-operative; PPV=Pars Plana Vitrectomy; SMD=Standardized Mean Difference; VEGF=Vascular Endothelial Growth Factor; the bold items mean P<0.05.

Supplement. 4. The details of head-to-head comparisons of silicone oil tamponade.

Regimen of anti-VEGF agents (OR, 95%CI)

| **Pre-Op more than 14d** | 1.19 (-2.16,4.54) | 1.95 (-2.58,6.48) | 1.89 (-1.41,5.20) | 2.18 (-1.51,5.88) |
| --- | --- | --- | --- | --- |
| -1.19 (-4.54,2.16) | **Pre-Op 6 to 14d** | 0.76 (-2.97,4.49) | 0.70 (-1.38,2.79) | 0.99 (-1.67,3.65) |
| -1.95 (-6.48,2.58) | -0.76 (-4.49,2.97) | **At the end of PPV** | -0.06 (-3.16,3.05) | 0.23 (-2.99,3.46) |
| -1.89 (-5.20,1.41) | -0.70 (-2.79,1.38) | 0.06 (-3.05,3.16) | **Sham** | 0.29 (-1.38,1.96) |
| -2.18 (-5.88,1.51) | -0.99 (-3.65,1.67) | -0.23 (-3.46,2.99) | -0.29 (-1.96,1.38) | **Pre-Op 1 to 5d** |

95% CI=95% Confidence Interval; Pre-Op=Pre-operative; PPV=Pars Plana Vitrectomy; OR=Odds Risk; VEGF=Vascular Endothelial Growth Factor; the bold items mean P<0.05.

Supplement 5. The details of head-to-head comparisons of intra-Op bleeding.

Regimen of anti-VEGF agents (OR, 95%CI)

| **Pre-Op more than 14d** | 0.41 (-2.70,3.51) | 1.19 (-2.22,4.60) | 2.34 (-2.31,6.99) | 2.57 (-0.49,5.63) |
| --- | --- | --- | --- | --- |
| -0.41 (-3.51,2.70) | **Pre-Op 6 to 14d** | 0.78 (-1.45,3.01) | 1.93 (-1.93,5.79) | ***2.16 (0.51,3.81)**** |
| -1.19 (-4.60,2.22) | -0.78 (-3.01,1.45) | **Pre-Op 1 to 5d** | 1.15 (-2.00,4.30) | 1.38 (-0.13,2.90) |
| -2.34 (-6.99,2.31) | -1.93 (-5.79,1.93) | -1.15 (-4.30,2.00) | **At the end of PPV** | 0.23 (-3.27,3.73) |
| -2.57 (-5.63,0.49) | ***-2.16 (-3.81,-0.51)**** | -1.38 (-2.90,0.13) | -0.23 (-3.73,3.27) | **Sham** |

95% CI=95% Confidence Interval; Intra-Op=intra-operative; Pre-Op=Pre-operative; PPV=Pars Plana Vitrectomy; OR=Odds Risk; VEGF=Vascular Endothelial Growth Factor; the bold items mean P<0.05.
